# Norbert F. Schneider and Michaela Kreyenfeld (eds.): Research Handbook on the Sociology of the Family. Cheltenham, UK: Edward Elgar Publishing. 2021

**DOI:** 10.1007/s10680-024-09706-6

**Published:** 2024-06-28

**Authors:** Christine Schnor

**Affiliations:** UCLouvain, Ottignies-Louvain-la-Neuve, Belgium

The “Research Handbook on the Sociology of the Family” edited by Schneider and Kreyenfeld was published in 2021 by Edward Elgar in their series of handbooks that are intended to be unique in their research focus, span disciplines and encourage “aspirations to pave the way for future research.” This aim was well met by Schneider and Kreyenfeld, who assembled 41 well-known international experts to provide readers information on key concepts, recent trends, cutting-edge methods, state-of-the-art discussions on classical themes and emerging topics, as well as theoretical debates relevant for understanding and researching contemporary European families.

The editorial Introduction (Part 1) is followed by 26 chapters organized in 7 parts. Part 2 discusses theoretical advances in family research, emphasizing the laboratory character of the European context for studying the link between institutional factors and demographic behavior. Neyer discusses the welfare-state approach and traces the place of family and gender issues in Welfare Regime Research; Nauck critically reflects on cross-cultural approaches to studying families; Widmer introduces the reader to the definition of family from a configurational perspective that goes beyond the classification based on household composition, understands family as a dynamic entity of individuals, and focuses on the interdependence among family members to document family diversity; Konietzka and Kreyenfeld address the link between the life course and the social structure, also describing the history of the life course approach.

Part 3 proposes new data perspectives in family research, encompassing digital data sources (Legewie and Fasang), qualitative longitudinal data (Bernardi), and network data (Viry and Herz). Part 4 focuses on family diversity and family change for example, Ehmer provides a historical perspective on the diversity of family forms, gender roles, and intergenerational family relations, going back to pre- and early industrial Europe. Sobotka and Berghammer document trends in union arrangements, fertility and childlessness, and the family context of reproduction. Liu and Esteve present age-specific patterns in living arrangements for working ages, distinguishing between living with parents, with a spouse, with children, or alone. Dykstra extends this discussion to later life.

Part 5 emphasizes the elements to understand specific family transitions in the life course, such as partner choice (Van Bavel), family dissolution (Mortelmans), and childbearing (Berrington), also for European migrant populations (Andersson). Part 6 touches on different aspects of relationships within families from demographic and sociological perspectives including relationships between grandparents and grandchildren (Skopek), between minor children and separated parents (Ulrike Zartler), and between romantic partners (Lenz and Adler). Part 7 discusses the gendered division of paid and unpaid work in families by providing a description of the main trends over the past decades and across European countries (Matysiak and Cukrowska-Torzewska) and a literature review on the topic (Sullivan) as well as by zooming into the lives of dual-earner couples (Grunow) and same-sex families (Evertsson, Eriksson, Kirsch, and Geerts). Finally, Sigle critically discusses fundamental theoretical concepts that aim to explain gendered work and care patterns.

The final section (Part 8) introduces some additional topics in family research. Social stratification in family behavior is a cross-cutting theme in this handbook. Bradshaw and Nieuwenhuis contribute to this discussion by examining families’ poverty risks and exploring how social policies can mitigate these risks. Then, Passet-Wittig and Bujard provide an overview on the different treatments of medically assisted reproduction available in Europe, discussing how these are experienced by parents and children and illustrate the trends in uptake. Finally, Merla, Kilkey Wilding, and Baldasar close this handbook with a discussion of the networks within transnational families.

This handbook is certainly a good choice for anybody looking for an up-to-date description of trends in family sociology, a critical discussion of central concepts, or a synthesis of the ongoing debates in the literature. In addition, it allows the reader to discover new perspectives in family sociology and offers a fresh and rich list of recommendations for future research. This handbook is also a very useful source for undergraduate students or doctoral students, as it provides a thorough and comprehensive guidance on core topics in European family sociology. I thus believe that students, researchers, and university teachers alike may find this handbook in many ways a very useful reference. The well-balanced combination of chapters on landmark theories, major trends, innovative methods and data, and key topics makes this book more than the sum of its parts.

Due to the limitations of space, it is not feasible to provide a comprehensive analysis of each chapter. Instead, I will focus on the chapter that I consider to be the most significant and discuss it in greater detail: the chapter by Wendy Sigle, in which she critiques “theoretical blind-spots,” i.e., the shortcomings of existing theoretical frameworks in the study of gender and family dynamics. For instance, she posits that these frameworks frequently neglect to consider the roles played by fathers within the family, thereby resulting in an “inattention” to their involvement. Taking the perspective of an imaginary young female student in the mid-twentieth century who wants to learn about social change, she describes how the “canonical knowledge and the conceptual toolbox” has shaped our way of doing research, of interpreting the world, and of setting ideals. For example, “The legacy of structural-functionalism and modernization theory can be detected in the persistent conceptual division of the (public) political economy from the (private) family” (p. 389) and in the idea of social change as developmental stages toward a defined institutional equilibrium. Sigle invites the reader to critically reflect on how men and women have been studied in family sociology and explains the reasons for lacking conceptual tools to explain the varying behaviors of men in the family. She takes Gary Becker’s theoretical model that emphasizes the advantages of a couple’s specialization in paid/unpaid work as a reference point to reflect on biases in research. For example, the implicit assumption that men always specialize in paid work has led researchers to study variations in women’s paid work much more than variations in men’s unpaid work as a factor in divorce. Sigle also criticizes that the concentration of contemporary research on the working mother has led “to a narrow, employment-focused conceptualization of gender equality” (p. 394) in which both men and women are expected to be full-time workers and societies are judged against this norm. In the same vein as this chapter, the handbook’s editors criticize in their introduction the absence of novel theories, which implies that researchers continue to refer to theoretical concepts that were developed decades ago.

This handbook is very rich in the topics covered, and, as the editor’s note, “such a handbook can never be complete.” Nevertheless, I have some critical reflections. The handbook mainly concentrates on the first parts of the life course (i.e., the First Age (childhood) and Second Age (young and middle adulthood)) and thus later life appears more as an add-on, but I believe it would deserve more attention. I would have preferred to see a more integrated discussion of the later parts of an individual’s life course (i.e., the Third Age (the ‘young-old’ retired and healthy adults) and Fourth Age (the ‘old-old’ and frail adults) for two main reasons. First, to avoid a narrow definition of the family as a unit which includes minor children. This definition limits the role of older persons mainly to that of a grandparent, which excludes childless persons from the picture and disregards the multiple positions older persons occupy as parents of adult children, spouses, siblings, or children of frail parents. Second, to account for the fact that European countries are aging societies, which means that the proportion of individuals in Third and Fourth Ages will continue to increase in the population, but also within families. This shift has implications for the sociological study of the family and deserves our attention. Another topic I missed is a discussion of reproductive rights of men and women and how they relate to core concepts of the family and gender. For instance, the question of whether gay couples have access to adoption, whether single women can conceive a child with the help of medically assisted reproduction, or whether and how women can decide to keep a pregnancy or to abort touches upon individuals’ rights to have and form a family and reflects questions about what constitutes a family. Finally, in the data section (part 3), I would have appreciated a discussion and critical reflection of the recent developments in the use of administrative data. These aspects may be covered in a future volume.

In sum, I believe that many chapters of this open-access handbook will turn into core references used in research and teaching.

